# Is *Encephalitozoon cuniculi* of Significance in Young Dogs With Neurological Signs?

**DOI:** 10.3389/fvets.2021.678968

**Published:** 2021-05-12

**Authors:** Tamar S. de Boer, Montse M. Diaz Espineira, Paul J. J. Mandigers

**Affiliations:** Department of Clinical Sciences, Faculty of Veterinary Medicine, Utrecht University, Utrecht, Netherlands

**Keywords:** cerebrospinal fluid analysis, dog, *Encephalitozoon cuniculi*, fenbendazole, infection, treatment 2

## Abstract

*Encephalitozoon cuniculi* is a microsporidium belonging to the phylum Microspora. A few reports have described the clinical significance of *E. cuniculi* infection in young dogs. In American and Japanese household dog populations, the seroprevalence was found to be 21%, indicating its wide-spread existence. To evaluate the clinical significance of *E. cuniculi* in a cohort of young dogs with neurological signs, the presence of the organism and possible response to treatment were studied. Over a 1-year period, all young dogs (<3 years old) that were referred with neurological signs were examined for the presence of *E. cuniculi*. Dogs were selected if serology revealed a clearly elevated IgM titer (>100) and/or if an EDTA-blood sample and/or urine sample tested positive by polymerase chain reaction (PCR). Sixteen dogs with various neurological signs were included in this study. Additional work-up included magnetic resonance imaging and cerebrospinal fluid analysis, but these revealed no abnormalities or indication of infection. All dogs were treated with fenbendazole for 10–30 days. Neurological signs disappeared completely in five dogs, 11 dogs continued to show neurological signs, and five dogs deteriorated and were euthanized, after which necropsy was performed in three. At necropsy no evidence of an *E. cuniculi* infection was found. We concluded that, although IgM titers and PCR indicated an *E. cuniculi* infection, it is most likely of limited clinical significance in young dogs.

## Introduction

*Encephalitozoon cuniculi* is a microsporidium belonging to the phylum Microspora (1, 2). *E. cuniculi* is an obligate intracellular parasite that can infect mammals, including humans, in which it is considered an opportunistic infection (2–4), but it is most frequently seen in rabbits (4). Mammals can get infected by ingestion of contaminated water or food, inhalation of spores or through *in utero* transmission (1, 5). The spores infect enterocytes and spread through blood or the lymphatic system. Predilection organs are among others lung, liver, kidney and brain (1, 5). It takes 3–5 weeks for the life cycle to complete. There are three known strains of *E. cuniculi*. Strain I was isolated from rabbits, strain II from rodents, and strain III from dogs (6).

Infected rabbits do not always show clinical signs, as a latent infection is possible. If clinical signs are seen in rabbits, these signs are subdivided into neurological signs, renal issues, ophthalmic problems, or combinations of these. The neurological signs are characterized by vestibular disease (torticollis, ataxia, rolling, and paresis). The renal form is characterized by chronic renal failure and its sequalae, such as weight loss, polyuria, polydipsia, and/or urinary incontinence. The ophthalmologic form is characterized by intraocular lesions, including cataract, uveitis, and hypopyon (5).

It was first observed that this agent could affect dogs in 1952, when Plowright (7) reported a granuloma containing the parasite in the brains of two puppies (7). Although the number of reports of *E. cuniculi* in dogs is small, these reports concern young or immunocompromised dogs. The clinical signs observed are the same as those reported in rabbits and are renal (4), ophthalmologic (8), and/or neurological in nature (9). The neurological signs described are blindness, ataxia, and seizures (9, 10).

Previous studies have demonstrated infections with the parasite in dogs. In both an American (11) and a Japanese population (12) of dogs, the seroprevalence for *E. cuniculi* was 21%. It has been suggested that adult dogs do not develop clinical disease after infection with *E. cuniculi*, because they are believed to be immunocompetent (13), but this does not apply to young dogs.

Although *E. cuniculi* has been identified in a number of studies as a possible cause of neurological signs in young dogs, it is not routinely tested in the first line and referral clinic. However, this is based on the earlier studies, remarkedly as it may be a differential diagnosis in young dogs. This study evaluated the clinical significance of *E. cuniculi* in a cohort of young dogs with neurological signs, by investigating its presence and possible response to a fenbendazole treatment.

## Materials and Methods

### Dogs

Over a period of 1 year, all young dogs (<3 years of age) that were referred to the Department of Clinical Sciences at the Faculty of Veterinary Medicine, Utrecht University, The Netherlands, were examined for the presence of *E. cuniculi*. Dogs were considered eligible for inclusion if they showed neurological signs, such as epilepsy, generalized ataxia, tremors, or dyskinesias in which an infection would be a logical differential diagnosis. All dogs were evaluated by a single investigator (PJJM).

### Procedure

After obtaining informed consent from each owner, a normal diagnostic work-up was performed. In all dogs, a complete hematology and biochemistry panel (University Veterinary Diagnostic Laboratory; www.uvdl.nl) as well as serology for toxoplasmosis and neospora (Veterinary Microbiology Diagnostic Centre; www.uu.nl/vmdc) was performed. *E. cuniculi* was evaluated by obtaining additional serum, EDTA-blood, and urine samples; these samples were examined for the presence of relevant antibodies (IgM and IgG) and of parasite antigen by means of an immunofluorescence antibody test (IFAT) and polymerase chain reaction (PCR), respectively (see section Treatment and Response to Treatment), at a commercial veterinary laboratory (European Veterinary Laboratory [EVL]; www.evlonline.org).

Based on the neurological signs and neuro-localization, all dogs underwent additional diagnostic steps, such as magnetic resonance imaging (MRI), cerebrospinal fluid (CSF) analysis, electromyographic studies (EMG), and/or specific DNA testing, if indicated. If any abnormality was found that was indicative for a specific treatment (for instance a dog with an atlanto-axial instability or a hydrocephalus), the dog was not eligible for this study.

### Inclusion and Exclusion Criteria

Dogs were only included if the routine biochemistry was free of abnormalities, if toxoplasmosis and neospora titers were found to be negative, and if additional work-up (CSF, MRI, DNA testing, etc.) did not exclude an infection as a possible cause of the neurological signs. Hence, if the MRI and/or CSF analysis was indicative of an inflammatory disorder, the dog was eligible for this study.

Dogs were only included and defined as possibly infected with *E. cuniculi* if serology showed a clearly elevated IgM titer and/or if the PCR of the EDTA sample and/or urine sample was found to be positive. The IgM was considered elevated if the value was 100 or more, while a titer of <30 was regarded as negative.

### Treatment and Response to Treatment

Included dogs were treated with fenbendazole only, at a dose of 50 mg/kg bodyweight, for a period of 10 days, after which the clinical outcome was evaluated (4, 14). If a dog did not respond to treatment, the dog was considered to be a non-responder. At this stage, the owner was offered a second treatment, or an additional treatment based on the presumed diagnosis. For instance, an anti-epileptic if the dog presented epilepsy, glucocorticosteroids if an immune-mediated process was suspected, etc. If an owner elected a second or third treatment, the dog's clinical outcome was again evaluated after 10 days. If a dog deteriorated regardless of the additional treatment and the owner elected euthanasia, a post-mortem examination was requested.

### Analytic Method

The three strains of *E. cuniculi* were grown on Vero or MRC cells using RPMI 1640 medium under 5% CO_2_. After cultivation, the spores were harvested, collected, concentrated, washed, and brought to a concentration of 10^9^, after which they were suitable for use in IFAT and a soluble ELISA antigen tests (Elisa-kit product-nr LA1003-AB01 from the EVL Laboratory; www.evlonline.org). The ELISA antigen was titrated on Greiner of Costar strip plates after production using a checkerboard titration, with known high (~1,350) and low titer (~450) sera and anti-rabbit horseradish peroxidase (from the EVL laboratory; www.evlonline.org; Cat: Do RA IgG-HRPO RTU19-05). After determining the optimal coating dilution, ELISA plates were coated and blocked, stabilized, and stored. A mixed coating was used for standard ELISA research. As a substrate, a 2-component substrate RTU from the EVL laboratory (www.evlonline.org) was used (Cat: TMBA-2019-06 and TMBB-2019-06 from the EVL Laboratory). The reaction was allowed to proceed for 10 min at 21°C; then, the reaction was stopped by adding 50 μl of diluted H_2_SO_4_, and the absorbance of the colored solution was read at 450 nm.

To assess the results, a sample ratio (positive control vs. standard method) or cutoff titer (TG-ROC plot) and 95% confidence interval was used. Batch validation was performed with pools of wild-type infected, type-selected, lyophilized sera (10^20^). To test field samples, two HRPO-conjugates of IgM and IgG and different controls were used. IgM determinations were only made after pre-absorption of IgG. Titrations (100, 300, 900, 2700) of the obtained samples were performed with IgM, starting at 1:50.

The PCR was based on an internal transcribed spacer and/or protein tyrosine phosphatase gene sequences (made by the EVL Laboratory and classified; www.evlonline.org). Conventional PCR was performed for type identification, while real-time PCR with ultra-black probes was also performed. Lyophilized infected cell cultures were used as controls at two levels of two different types of PCRs (standard methods, from Jena Bioscience, Jena, Germany).

### Statistical Analysis

Results were statistically analyzed with IBM SPSS Software, version 24 (IBM, Chicago, IL, USA). Descriptive statistics and a Pearson chi-squared test were performed. Results were considered significant when the *p*-value was <0.05.

## Results

### Dogs

Forty-two young dogs (<3 years of age) were eligible for this study, but only 16 met the final inclusion criteria. The dog breeds varied, with no breed overrepresented. The age of onset had a median of 8 months (range 3–39 months). The duration of illness had a median of 1 month (range 1–15 months) (Table 1).

**Table 1:** Included dogs, age in months at the time of referral, and the duration of neurological signs present.

**Case number**	**Breed**	**Age (months)**	**Illness duration (months)**	**Neurological signs**
1	Labrador Retriever	32	1	Head tremor, vestibular signs
2	Labradoodle	28	2	Generalized tremors and ataxia
3	Dutch Shepherd	4	1	Head tremor, epileptic seizures
4	French Bulldog	8	1	Head tremor, epileptic seizures, generalized ataxia
5	Cesky Fousek	39	1	Head tremor, epileptic seizures, generalized ataxia
6	German Shepherd	4	1	Tonic-clonic seizures
7	Pointer	33	7	Paroxysmal dyskinesia
8	Pug	19	10	Head tremor, vestibular signs
9	Wetterhoun	3	1	Generalized ataxia, vestibular signs
10	Old English Bulldog	23	4	Head tremor, epileptic seizures
11	Nova Scotia Duck Tolling Retriever	3	6	Tremor front legs, ataxia of hindlegs
12	Jack Russel Terrier	4	1	Head tremor, epileptic seizures
13	Norfolk Terrier	7	1	Abnormal behavior, epileptic seizures
14	Tervueren Shepherd	8	1	Ataxia of hindlegs
15	Border Collie	8	2	Generalized ataxia, vestibular signs
16	Rottweiler	24	15	Ataxia of hindlegs

### Neurological Signs

Neurological signs in 14 of the 16 dogs were suggestive of at least an intracranial neuro-localization (Table 1). Twelve of the 16 dogs had multiple varied neurological signs, including head tremor (seven dogs), epileptic seizures (focal or generalized, in seven dogs), generalized tremors (two dogs), vestibular signs (four dogs), and generalized ataxia (five dogs). Only four dogs showed a single neurological sign: one with only tonic-clonic seizures, one with paroxysmal dyskinesia, and two with ataxia of the hindlegs (Table 1).

### Laboratory Findings

Routine hematology and biochemistry revealed some abnormality in all dogs. Fourteen dogs had a positive PCR in their EDTA-blood sample, of which one also a PCR-positive urine sample (no other dog had a positive urine sample) (Table 2). Of these 14 dogs, four dogs had an elevated IgM titer of 100, all others had IgM titers below 30. Two of the 14 dogs had an IgG titer of 300, and seven a titer of 100; the remaining five had titers below 30. Two dogs had an elevated IgM titer (300 and 1,000), but a negative PCR for both urine and EDTA-blood sample. The IgG titer in these two dogs was 300. Both dogs showed signs of generalized ataxia, head tremor, and epileptic seizures (Table 2).

**Table 2:** Laboratory and additional findings and outcomes.

**Case number**	**IgM titer**	**IgG titer**	**PCR EDTA**	**MRI**	**CSF protein**	**Treatment period**	**Outcome**	**Pathology**
1	<30	<30	Positive	-		10 days	Improved	NA, alive
2	100	100	Positive	No abn.	0.3	20 days	Improved	NA, alive
3	<30	100	Positive	-		10 days	Improved	NA, alive
4	1,000	300	Negative	No abn.	0.35	10 days	Improved	NA, alive
5	300	300	Negative	-		30 days	Improved	NA, alive
6	100	100	Positive	No abn.	0.2	10 days	Slight improvement	NA, alive
7	30	100	Positive	No abn.	0.2	10 days	No improvement	NA, alive
8	<30	100	Positive	No abn.	0.28	20 days	No improvement	NA, alive
9	<30	30	Positive	No abn.	0.18	10 days	No improvement	NA, alive
10	30	100	Positive	-		10 days	No improvement	NA, alive
11	30	300	Positive	No abn.	0.3	10 days	No improvement	NA, alive
12	100	100	Positive	-		20 days	No improvement, died	Not allowed
13	<30	<30	Positive	-		30 days	No improvement, died	Not allowed
14	100	300	Positive	No abn.	0.33	20 days	No improvement, died	Yes, no abnormalities
15	30	30	Positive	-		10 days	No improvement, died	Yes, no abnormalities
16	<30	<30	Positive	No abn.	0.4	10 days	No improvement, died	Yes, no abnormalities

*Dogs included with their IgM, IgG titers, PCR results in the EDTA blood sample, as well as the MRI findings and CSF protein amount. The CSF cell count was not abnormal in all cases. Case number 14 also had a positive urine PCR. Additionally, the treatment period, clinical outcome, and if necropsy was performed, the outcome are also listed*.

### Additional Diagnostic Evaluation

In nine of the 16 dogs, an MRI scan and CSF analysis were performed. None of the MRI scans revealed any abnormality, and CSF was regarded to be normal in all nine dogs (Table 2).

### Response to Treatment

All dogs were treated according to the protocol. Five dogs responded to the treatment, of which two responded within 10 days, two within 20 days, and one during the third period of fenbendazole treatment. All five dogs had signs suggestive of a multifocal or generalized neurological disorder. Two of these responders had a negative PCR, but a clearly elevated IgM titer. Two had a positive PCR and elevated IgM and/or IgG, while one dog only had a positive PCR, but without IgM and IgG elevation.

Eleven dogs did not improve, regardless of treatment. All these dogs received a second course of fenbendazole, but as they did not respond, an additional treatment, based on the presumed diagnosis, was started. Only one out of these 11 dogs did respond after the additional treatment with an anti-epileptic. The condition of five of these 10 non-responding dogs deteriorated, and euthanasia was elected. Three of these dogs were necropsied, but in none of these cases was the histopathology abnormal, and there was no indication of an *E. cuniculi* infestation.

In all dogs, serology and PCR were repeated after either 10 (the five responding dogs) or after 20 days (the 11 non-responding dogs), and in all dogs the PCR was found to have become negative. There was no statistically significant difference for dogs that had improved compared to those that did not improve in terms of MRI findings (*p* = 0.39), CSF protein level (*p* = 0.43), or treatment outcome and period (*p* = 0.82).

## Discussion

Although some reports suggest that *E. cuniculi* is of significance in young dogs, it is not routinely tested. This is remarkable, as previous studies not only described the clinical signs in young dogs (8–10), but also a rather high seroprevalence of 21% in American (11) and Japanese (12) populations, suggesting that dogs do come into contact with the parasite. For this reason, we evaluated the clinical significance of this parasite in a group of young dogs. In rabbits with clinical signs, a diagnosis is considered confirmed if *E. cuniculi* spores are detected in the urine or if the parasite is found to be present in the blood or urine sample based on PCR (15). Whether this also applies to dogs is not known.

The 16 examined dogs were all found to be positive either by PCR (14 dogs) or by clearly elevated IgM titer (two dogs). The latter is suggestive of an immune response to the parasite, while the first demonstrates that the parasite was ingested. As most of our dogs had either an elevated IgM titer (6 of 16) or IgG titer (11 of 16), we concluded that there was an immune response to the parasite in these dogs. The clinical signs observed in the dogs were in accordance with previous studies (9, 10) and were in all dogs, except for four, suggestive of multifocal neuro-localization. In young dogs, the differential diagnosis would be intoxication, inflammatory, either infectious or immune-mediated, generalized or multi-focal neoplasia, and/or degenerative disorders. Based on the protocol followed, the most likely differential diagnosis in these dogs would be an inflammatory etiology.

Interestingly, the MRI scans and CSF analysis were normal in nine of the 16 dogs. There are no detailed reports describing MRI findings and CSF analysis in dogs with *E. cuniculi* infection. However, one report in a human case states that both CSF and MRI findings were clearly abnormal (16). Furthermore, the CSF analysis in rabbits diagnosed with *E. cuniculi* infection is also clearly abnormal with elevated cell counts as well as protein levels (17); this was not the case with the dogs in this study. Despite the negative CSF analysis, it would have been of interest to perform a PCR on the CSF for the various neurotropic infections. However, at the time of this study this test was not available.

Interestingly five dogs improved after the fenbendazole treatment. Four of these dogs suffered from head tremor, which is also classified as an idiopathic head tremor (18), and is known to resolve by itself in many cases. Nevertheless, all four these dogs also had other neurological signs, suggestive of a multifocal localization, and therefore the head tremor was less likely to be idiopathic. Eleven dogs did not respond to treatment, and an additional treatment had to be initiated. Despite this, five dogs showed deterioration of neurological signs and the owners elected euthanasia. Three of these dogs were necropsied but no abnormalities suggestive of an *E. cuniculi* infection were observed. In rabbits, the diagnosis is confirmed if granulomatous inflammation with spores is found in renal, hepatic, and/or brain tissue at post-mortem (5), as also described by Plowright (7) and Snowden et al. (10).

This study posed the question of whether *E. cuniculi* is a true pathogen in dogs. All dogs included into this study were infected with *E. cuniculi*, but MRI and CSF analysis revealed no abnormalities in nine of these dogs nor in the three necropsied dogs. And there was a lack of response to treatment in 11 dogs. Based on these results, *E. cuniculi* is most likely of limited clinical significance in young dogs.

This study has limitations. First of all, the prevalence of *E. cuniculi* in dogs and rabbits in the Netherlands is not studied and therefore unknown. *E. cuniculi* as a disease in rabbits is frequently observed in the Netherlands but exact morbidity and mortality figures are sadly unknown. Secondly the study population is small. It may be of interested to perform this study together with a prevalence study in other countries as well. And if *E. cuniculi* was the cause of neurological signs in these examined dogs, the lack of response could be because of our choice of fenbendazole treatment. Rabbits suffering from *E. cuniculi* can be treated with fenbendazole or albendazole (4, 14). However, the uptake of fenbendazole is less than that of albendazole, which may underlie the lack of response observed. However, albendazole is embryotoxic, teratogenic, and can cause serious side effects in dogs, and hence, we chose fenbendazole as it is registered as safe to be used in dogs (19).

## Conclusion

The parasite *E. cuniculi* was found to be present in 16 dogs presenting various neurological signs. However, MRI and CSF analysis were not found to be abnormal in nine dogs, three dogs were necropsy-negative, and there was a lack of response to treatment in 11 dogs. Based on the current results, *E. cuniculi* is most likely of limited clinical significance in young dogs.

## Data Availability Statement

The raw data supporting the conclusions of this article will be made available by the authors, without undue reservation.

## Ethics Statement

Ethical review and approval was not required for the animal study because all dogs were referred cases with neurological deficits. Written informed consent was obtained from the owners for the participation of their animals in this study.

## Author Contributions

TB performed this study as her master thesis. MD co-supervised and reviewed with PM the study. PM set up the study, examined all dogs, supervised, and reviewed the analysis. All authors read and approved the final manuscript.

## Conflict of Interest

The authors declare that the research was conducted in the absence of any commercial or financial relationships that could be construed as a potential conflict of interest.
